# Supplementary material for: Anticancer Activity and Molecular Mechanisms of an Ursodeoxycholic Acid Methyl Ester-Dihydroartemisinin Hybrid via a Triazole Linkage in Hepatocellular Carcinoma Cells
Source: Molecules. 2023 Mar 3;28(5):2358. doi: 10.3390/molecules28052358 (PMC10005781; doi:10.3390/molecules28052358)
Supplement: Supplementary file 1 [file molecules-28-02358-s001.zip › molecules-2217711-supplementary.pdf]

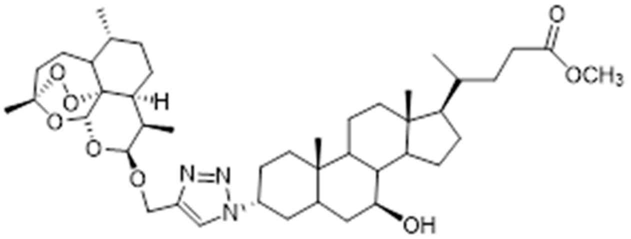

O
OCH3
H
N=N
N
OH

^{1}H-NMR.

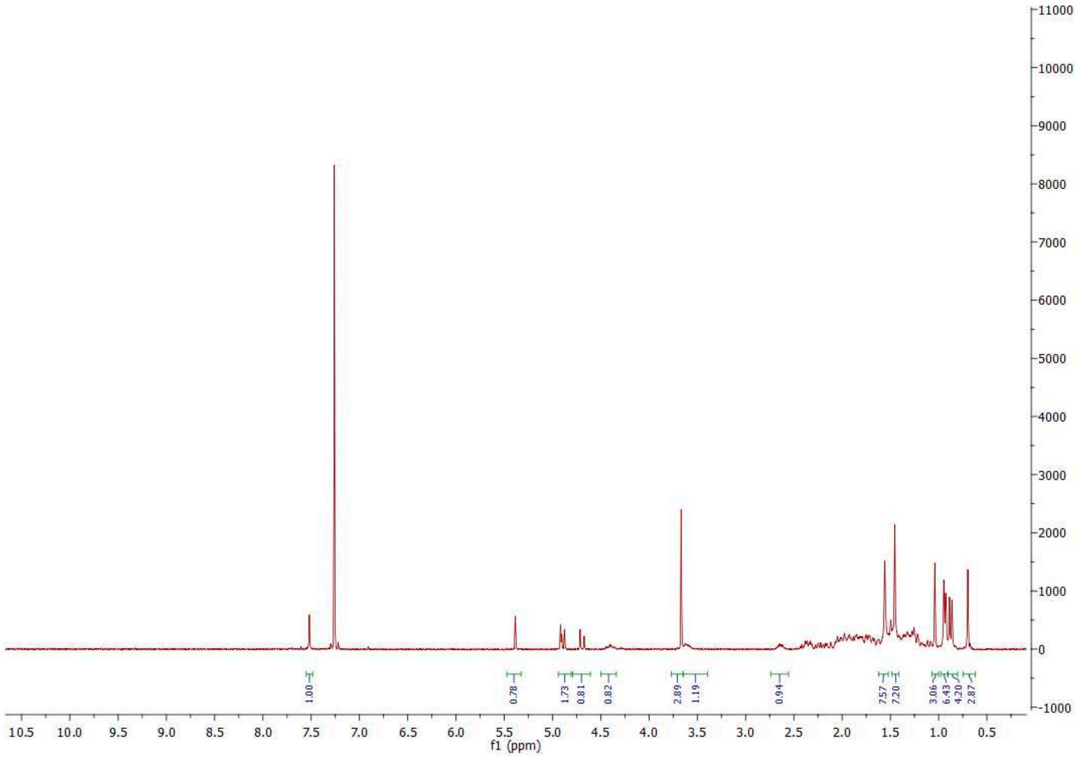

11000
10000
9000
8000
7000
6000
5000
4000
3000
2000
1000
0
-1000
1.00
0.78
1.73
0.81
0.82
2.89
1.19
0.94
7.57
7.20
3.06
6.43
4.20
2.87
10.5 10.0 9.5 9.0 8.5 8.0 7.5 7.0 6.5 6.0 5.5 5.0 4.5 4.0 3.5 3.0 2.5 2.0 1.5 1.0 0.5
f1 (ppm)

^{13}C-NMR

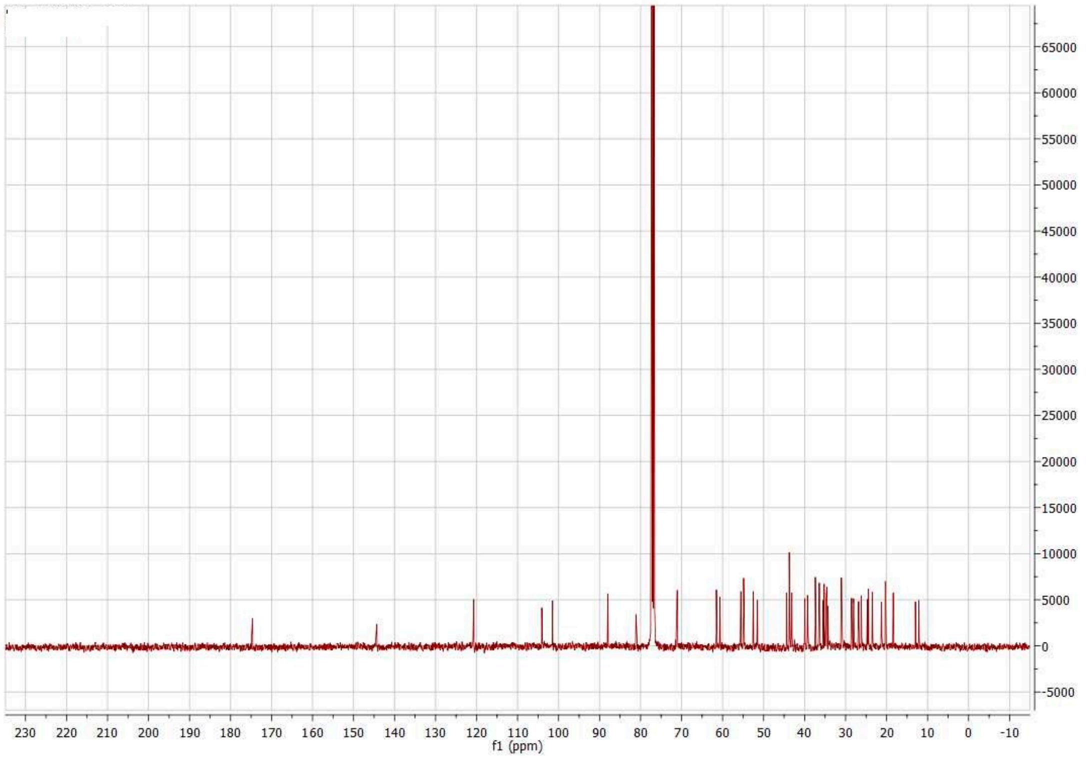

Figure S1. Spectroscopic data of compound UDCMe-Z-DHA: ^{1}H-NMR spectrum; ^{13}C-NMR spectrum. The crude product obtained as reported in a previous work [Ref. 19] was purified by flash chromatography on silica gel by elution with EtOAc/Cy 1:1 in presence of a pad of Florisil®. The desired product was obtained as a pure white powder. Analytical and spectroscopic data were in agreement with that reported previously [Ref. 19]. HRMS (ESI) (+) calcd for $C_{43}H_{68}N_3O_8$ $[M+H]^+$ 754.5001, found 754.4992.

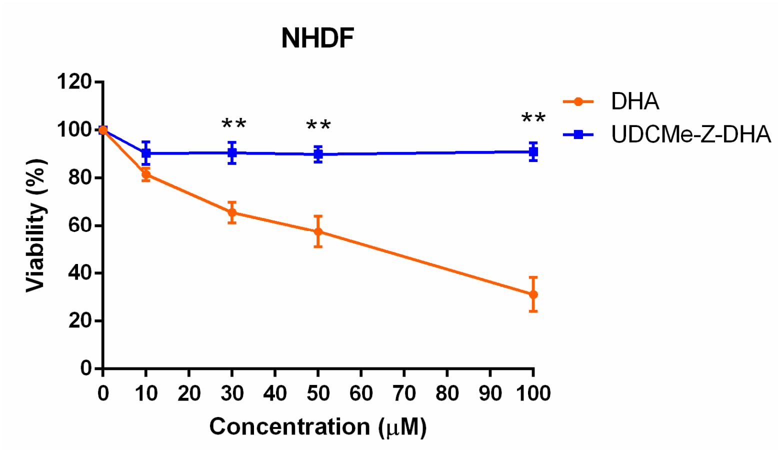

Figure S2. UDCMe-Z-DHA is less toxic than DHA toward normal human dermal fibroblast cells. Normal human dermal fibroblast (NHDF) cells were seeded in 96-well plates, treated with DHA or UDCMe-Z-DHA for 72 h, and cell viability was measured by the MTT assay. Data are presented as mean ± SEM of at least three independent experiments. Statistical significance was assessed by two-tailed Student's *t*-test. ** $p < 0.01$.

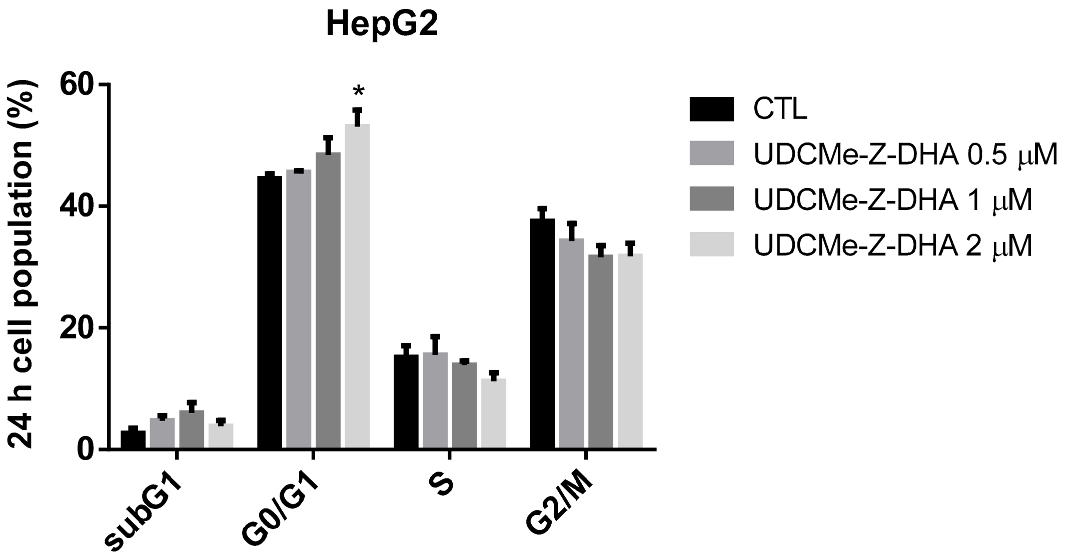

Figure S3. Cell cycle distribution of HepG2 cells treated with UDCMe-Z-DHA for 24 h. Cells were seeded in 12-well plates, treated with UDCMe-Z-DHA for 24 h, harvested by trypsinization, and subjected to PI staining and flow cytometric analysis. Data are presented as mean ± SEM of at least three independent experiments. Statistical significance was assessed by two-tailed Student's *t*-test. * $p < 0.05$.
